# Enhanced sunlight photocatalytic activity and biosafety of marine-driven synthesized cerium oxide nanoparticles

**DOI:** 10.1038/s41598-021-94327-w

**Published:** 2021-07-19

**Authors:** Somayeh Safat, Foad Buazar, Salim Albukhaty, Soheila Matroodi

**Affiliations:** 1 Department of Marine Chemistry, Khorramshahr University of Marine Science and Technology, P.O. Box 669, Khorramshahr, Iran; 2 Department of Chemistry, University of Misan, P.O. Box 62001, Maysan, Iraq; 3 Department of Marine Biology, Khorramshahr University of Marine Science and Technology, P.O. Box 669, Khorramshahr, Iran

**Keywords:** Nanoscience and technology, Nanobiotechnology, Nanoparticles

## Abstract

This contribution presents the biosynthesis, physiochemical properties, toxicity and photocatalytic activity of biogenic CeO_2_ NPs using, for the first time, marine oyster extract as an effective and rich source of bioreducing and capping/stabilizing agents in a one-pot recipe. CeO_2_ NPs formation was initially confirmed through the color change from light green to pale yellow and subsequently, their corresponding absorption peak was spectroscopically determined at 310 nm with an optical band-gap of 4.67 eV using the DR-UV technique. Further, XRD and Raman analyses indicated that nanoceria possessed face-centered cubic arrangements without any impurities, having an average crystallite size of 10 nm. TEM and SEM results revealed that biogenic CeO_2_ NPs was approximately spherical in shape with a median particle size of 15 ± 1 nm. The presence of various bioorganic substances on the surface of nanoparticles was deduced by FTIR and TGA results. It is found that marine-based nanoceria shows no cytotoxic effect on the normal cell, thus indicating their enhanced biocompatibility and biosafety to living organisms. Environmentally, due to energy band gap, visible light-activated CeO_2_ nanocatalyst revealed superior photocatalytic performance on degradation of methylene blue pollutant with removal rate of 99%. Owing to the simplicity, cost-effectiveness, and environmentally friendly nature, this novel marine biosynthetic route paves the way for prospective applications of nanoparticles in various areas.

## Introduction

In the last few years, biogenic nanoparticles have gained surging popularity in all fields particularly in medicine and water purification mainly owing to their safety, biocompatibility, and biological properties^1,2^. The notion “biogenic” refers to a variety of biosynthetic pathways based on flora and fauna extracts. These natural resources include sea cucumber^3^, algae^4,5^, plants^6,7^, bacteria, yeast and fungi microorganisms among others^8^. Due to their intrinsic capabilities, green methodologies facilitate the reduction of dissolved metal ions to zero valence state and eventually yield the pertaining nanoparticles. More specifically, marine resources hold the most promising area for the development of a new generation of the biological nanoparticle. It is envisaged that water cover 71% of the Earth's surface and 96.5% of all the Earth's water is associated with the oceans^9^, hence, the teeming world of naturally occurring microorganisms with complexity and diversity in the marine ecosystem could consider as an essential and readily available factory for manufacturing beneficial and economical nanoscale materials for numerous applications^10^. The thriving ocean environment is a rich source of bioactive compounds that increasingly have innovative contributions in wide pertinent of all viable areas such as biomedicine, biotechnology, food, and cosmetic product developments. Despite global growing intuitive concern on the substantial environmental detrimental facet, an appreciable number of researches yet focus on chemical and physical methods. Therefore, a comparatively inconsiderable proportion of the literature reports has allocated to the utilization of sustainable marine sources for the green synthesis of nanoparticles^11^.

The edible oysters (*Saccostrea cucullata*) as a seafood are marine invertebrates belonging to brackish bivalve molluscs. They are dweller of intertidal zones and rich in a myriad of bioactive substances including carbohydrates, polyphenols, peptides, vitamins (vitamins A and B12), minerals (calcium and zinc), and proteins^12^. Traditionally, oyster’s consumption whether food or medicine are prevailed in Asian countries especially those living in coastal regions^13^. It is estimated that around two billion oysters are consumed annually since they are nutritionally balanced source of food and impart great health benefits. As a valuable ocean resource of the blue economy, the bulkier rock oyster and Pacific oyster species commercially hold predominant contribution in therapeutic, food, cosmetics products, and hence, they are cultivated all year round.

In recent years, cerium oxide nanoparticles (CeO_2_ NPs, nanoceria) have been received momentous interest owing to their inimitable physiochemical properties, biocompatibility, and bioactivities^14,15^. They were broadly exploited in various fields such as therapeutics agents in acute kidney injury^16^, catalysis, drug delivery careers, and environmental pollution scavenger^17^. It is found that effective oxidative stress, free-radical scavengers’ role, and enzyme-mimetic catalytic performance of nanoceria mostly due to variation between the Ce^4+^ and Ce^3+^ cations proportion which on account of oxygen deficiencies in lattice arrangement of their crystal structure. The presence of oxygen vacancy sites, remarkably promote reduction–oxidation reactions of CeO_2_ NPs in living cell regimes and thus, render them highly effective therapeutics candidate in diagnoses and treatment a number of microbial pathogens and other neurodegenerative disorders^18^. On the other hand, the promoted biological properties of nanoceria are remarkably affected by the synthesis routes which in turn induce miscellaneous particle size, shape, and size distribution. In this connection, a number of chemical and physical synthetic pathways has been addressed the production of nanoceria^19^.

**Figure 1:**
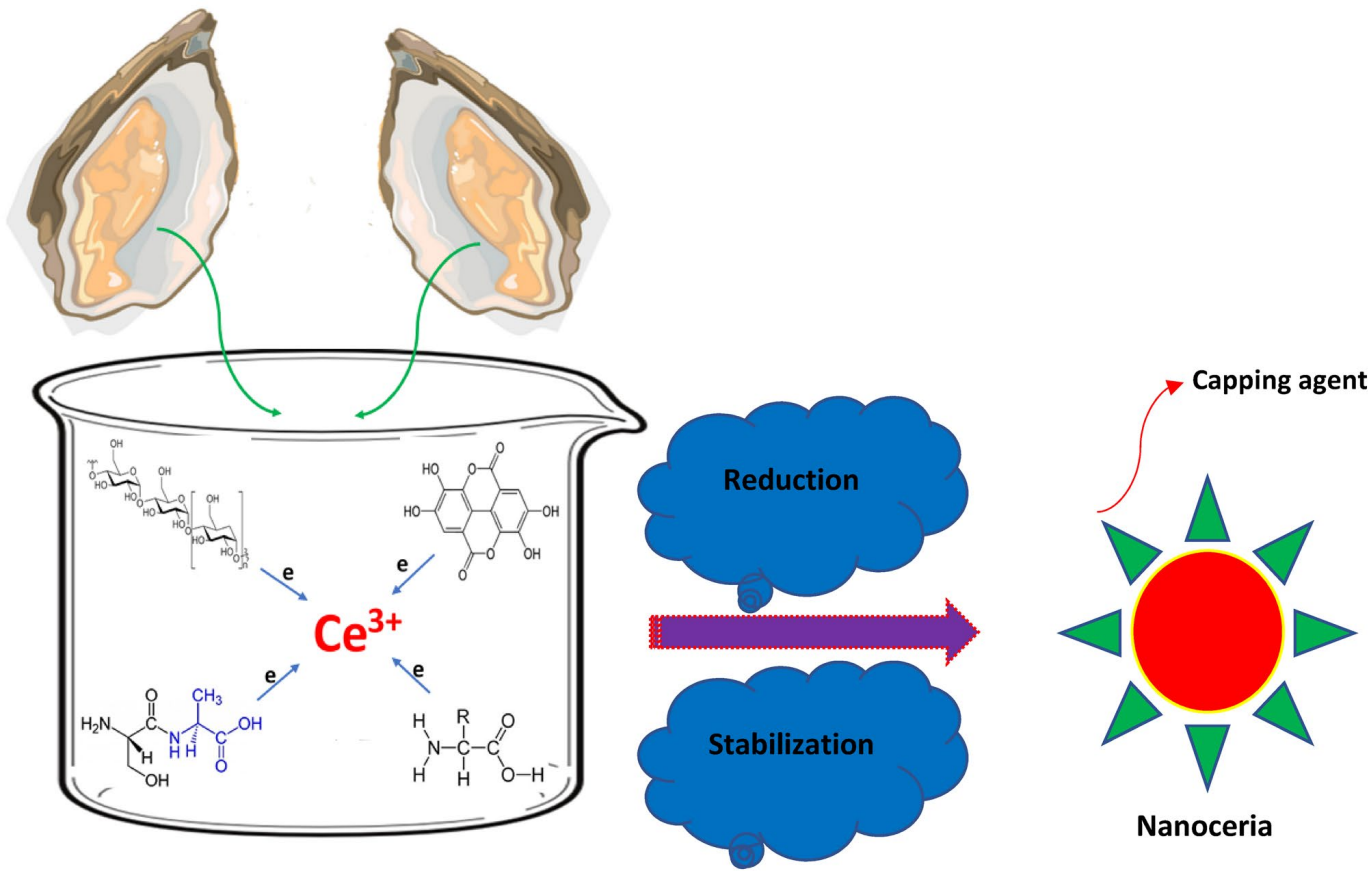
Schematic representation of the biosynthetic pathway of nanoceria using marine oyster extract.

In this study, we present a novel synthetic approach for the generation of CeO_2_ NPs utilizing marine oyster extract as a facile, cost-effective, and eco-friendly platform of which ample phytochemicals play multiple roles in reduction, stabilization, and fine distribution of nanoceria through oxidation–reduction (redox) reactions (Fig. 1). The relevant physicochemical characterization influencing the biological synthesis of marine-mediated CeO_2_ NPs are scrutinized and elucidated as well. The cytotoxicity and photocatalytic properties of green nanoceria have investigated against healthy cells and toxic cationic methylene blue dye in wastewater samples.

## Result and discussion

### UV–Vis analysis

**Figure 2:**
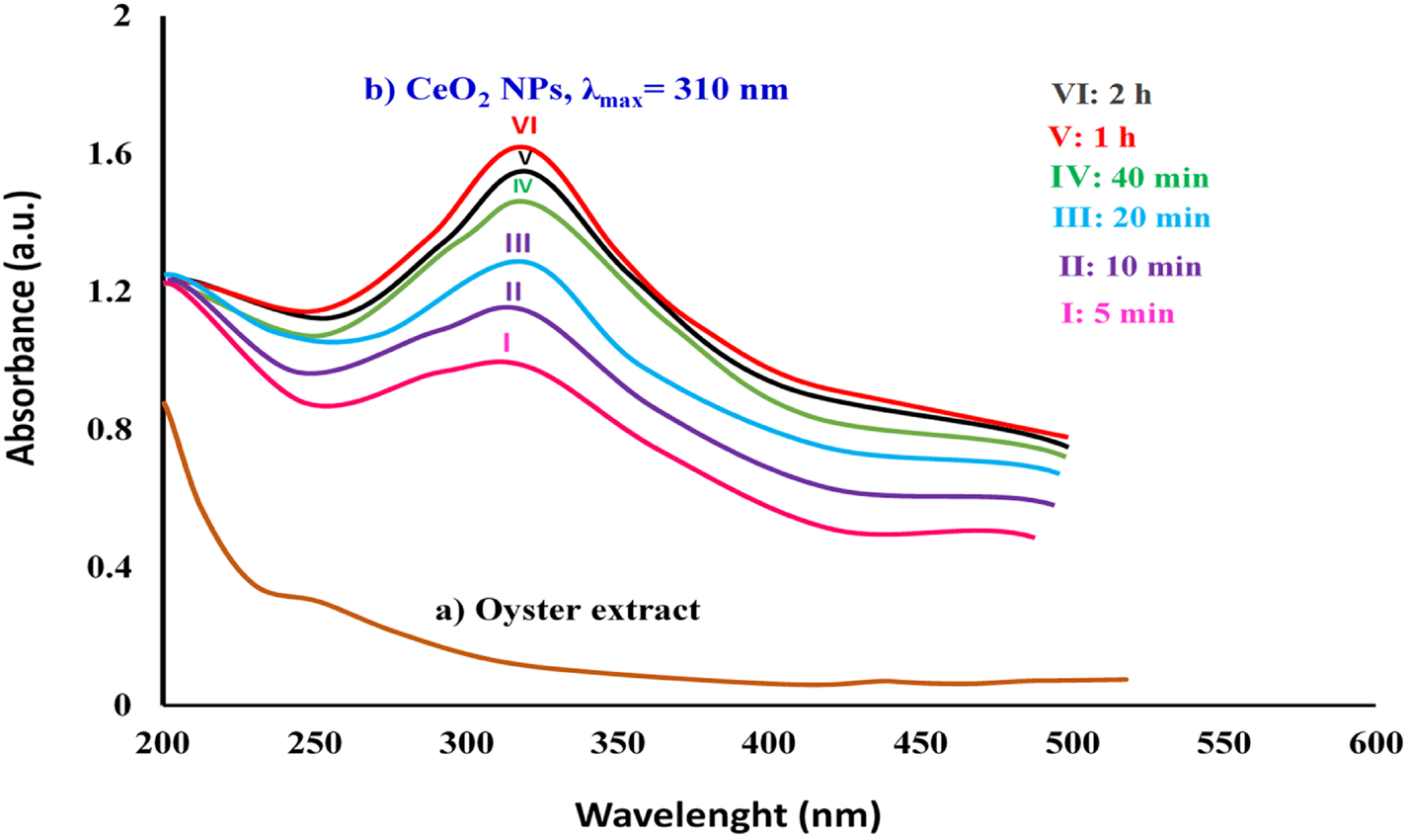
UV–Vis spectra of (**a**) raw oyster extract and (**b**) bioprepared CeO_2_ NPs as function of time.

**Figure 3:**
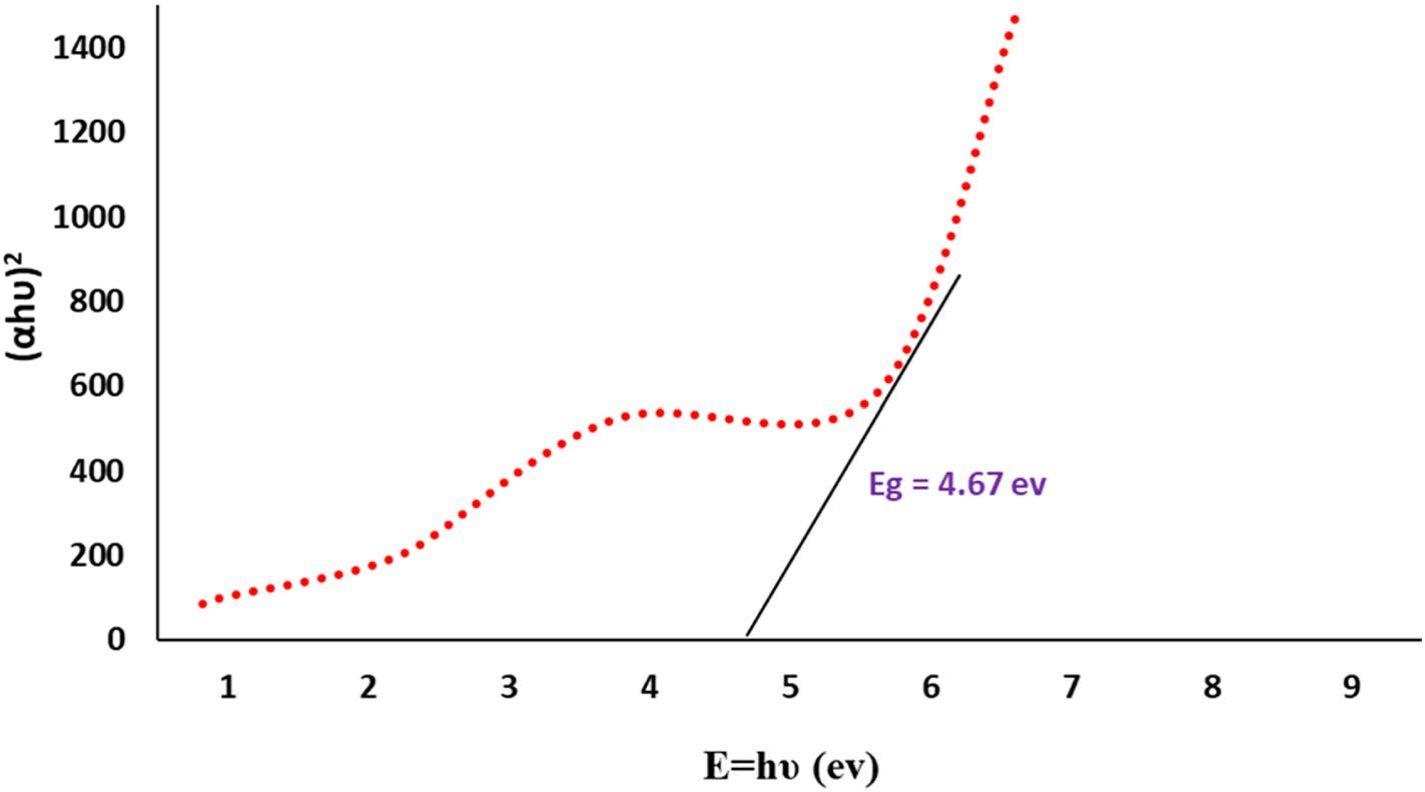
Tauc plot of biosynthesized CeO_2_ NPs using marine oyster extract.

The primary visual observation of CeO_2_ NPs formation was discerned when the solution color changed from light-green to pale yellow slurry^20^. Further, the confirmation of optical absorption behavior of the bioproduced nanoceria powder was ascertained in the Diffuse Reflectance UV–Vis spectroscopy showing a sharp absorbance peak at 310 nm (Fig. 2a,b). The rate of a reaction attributed to bioreduction of cerium cations and production of nanoparticles was monitored using a fixed wavelength UV–Vis spectrophotometer as a function of a variable time interval (5 min, 10 min, 20 min, 40 min, 1 h, 2 h). The results revealed that after 40 min the reaction reached the fullest and hence it was completed appearing no significant change beyond this time in peak position or intensity, as illustrated in Fig. 1b. In a similar study, Sarani and Miri demonstrated a comparable absorption peak at 317 which was assigned to the biosynthesized CeO_2_ NPs using the aqueous extract of *Prosopis farcta*^21^. Using the Tauc equation $, {\left(\alpha hv\right)}^{2}={\left(A\right)}^{2}{\left(hv-Eg\right)}^{2}$, the direct optical band-gap (Eg) of the biosynthesized CO_2_ NPs was estimated to be 4.67 eV which is remarkably higher than the corresponding bulk cerium oxide value (3.19 eV) (Fig. 3)^22^.

### XRD characterization

**Figure 4:**
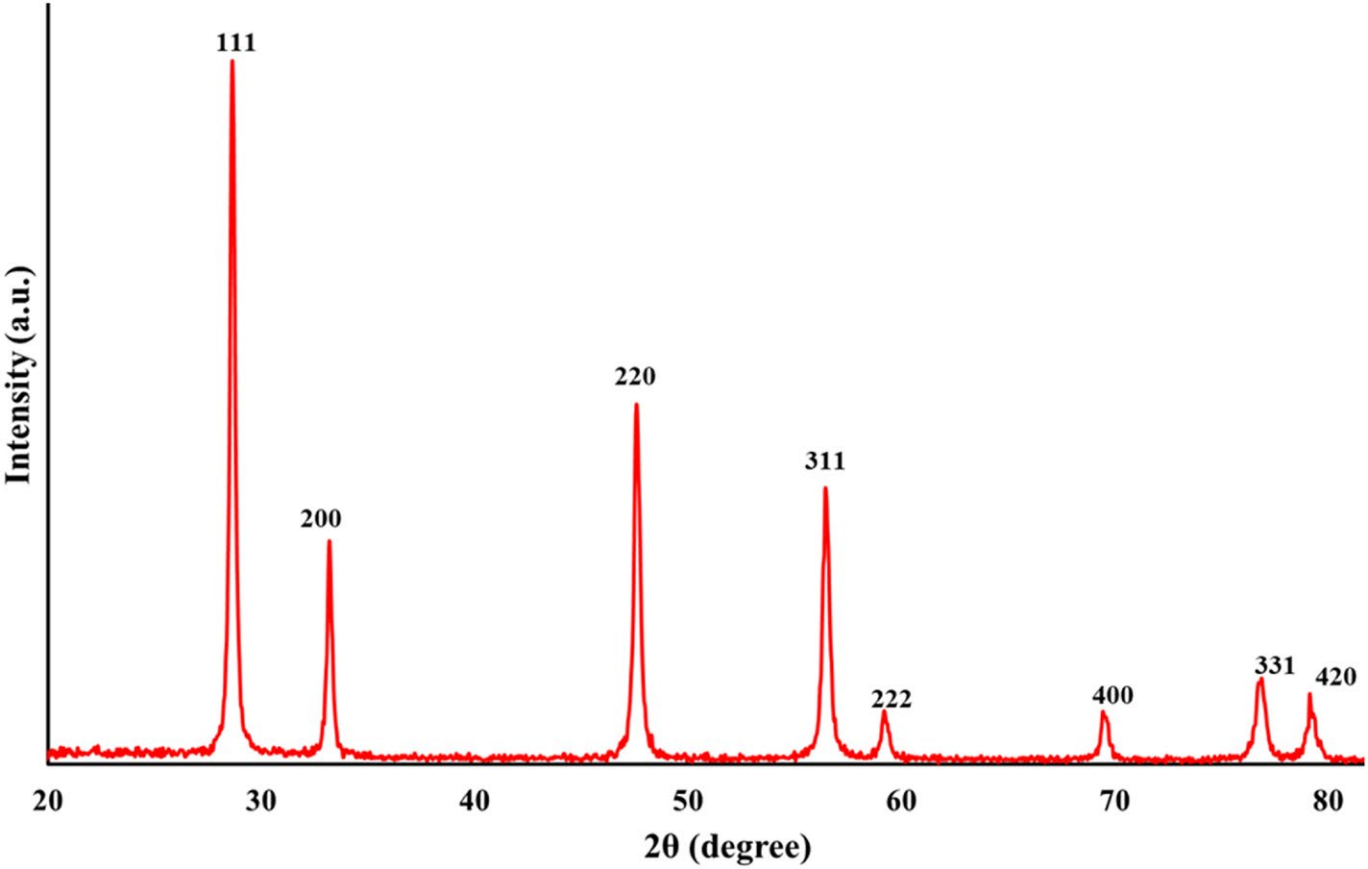
XRD pattern of the marine Oyster assisted biosynthesized CeO_2_ NPs.

Figure 4 illustrates the XRD pattern of biofabricated CeO_2_ NPs. The presence of eight diffraction peaks corresponds to (111), (200), (220), (311), (222), (400), (331), and (420) crystallographic planes are precisely well matched to Joint Committee on Powder Diffraction Standards (JCPDS) (card no 96-900-9009). The obtained crystal lattice demonstrates that biogenic CeO_2_ NPs possess a single-phase cubic fluorite structure where each cerium site is occupied by eight oxygen sites in a face-centered cubic fashion (a = b = c = 5.14, α = β = γ = 90°). In an akin research, Maqbool et al*.*, reported that biosynthesized CeO_2_ NPs by *Olea europaea* leaf extract shows a single face cubic center (fluorite structure)^23^. Moreover, no additional peaks were observed in the XRD pattern, indicating the purity of bioproduced nanoparticles. Using Debye–Scherrer equation, and from the most intensive broad Bragg peak at 28.54°, the approximate crystallite size of CeO_2_ NPs was found to be 8.42 nm.

**Figure 5:**
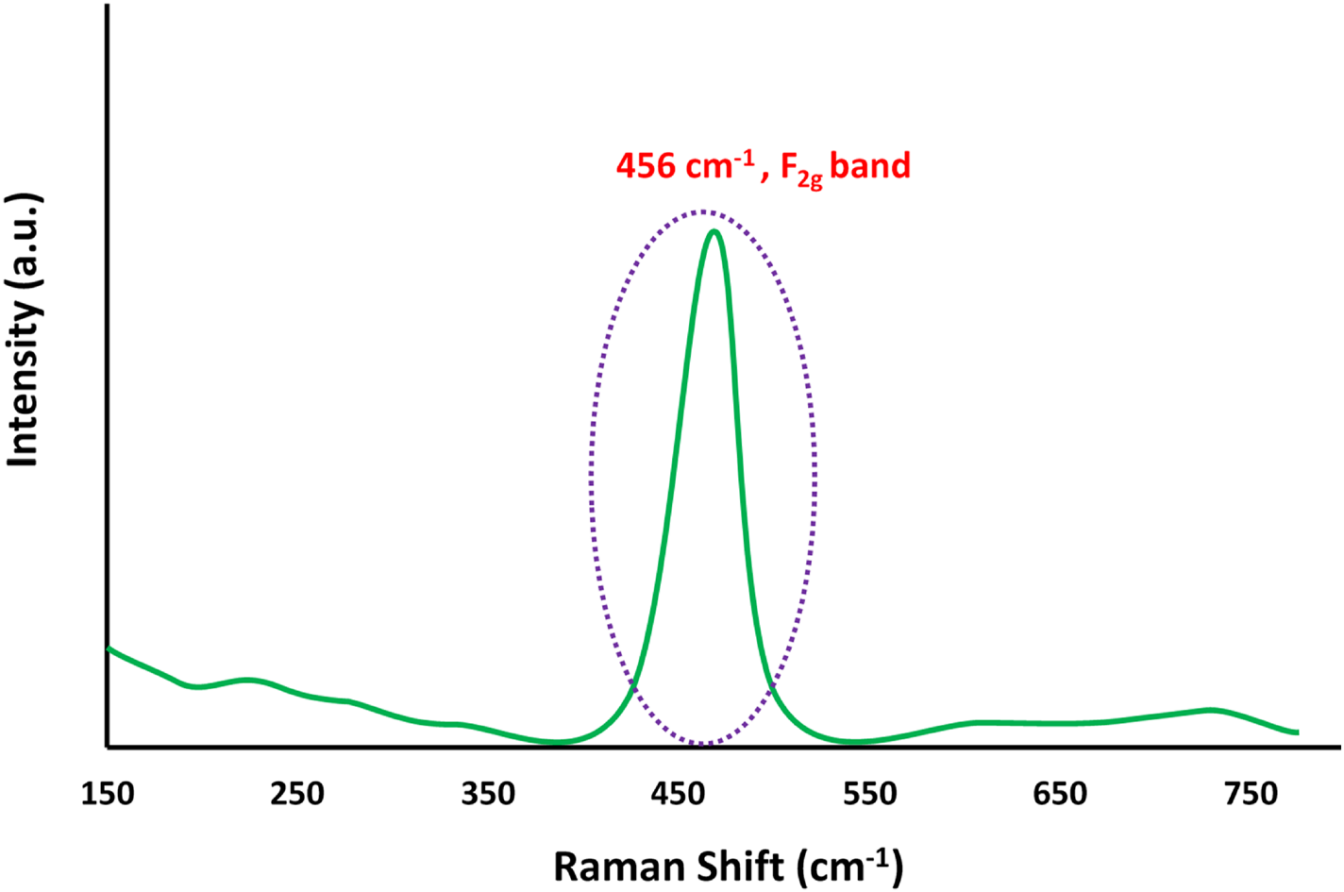
Raman spectrum of biofabricated CeO_2_ NPs using marine oyster extract.

Correspondingly, the Raman spectrum of nanoceria depicted a noticeable peak at 456 cm^−1^ which is may attribute to the F_2g_ Raman active mode, confirming cubic fluorite crystal structure of biogenic nanoceria, supporting the XRD results (Fig. 5)^24^.

### FTIR analysis

**Figure 6:**
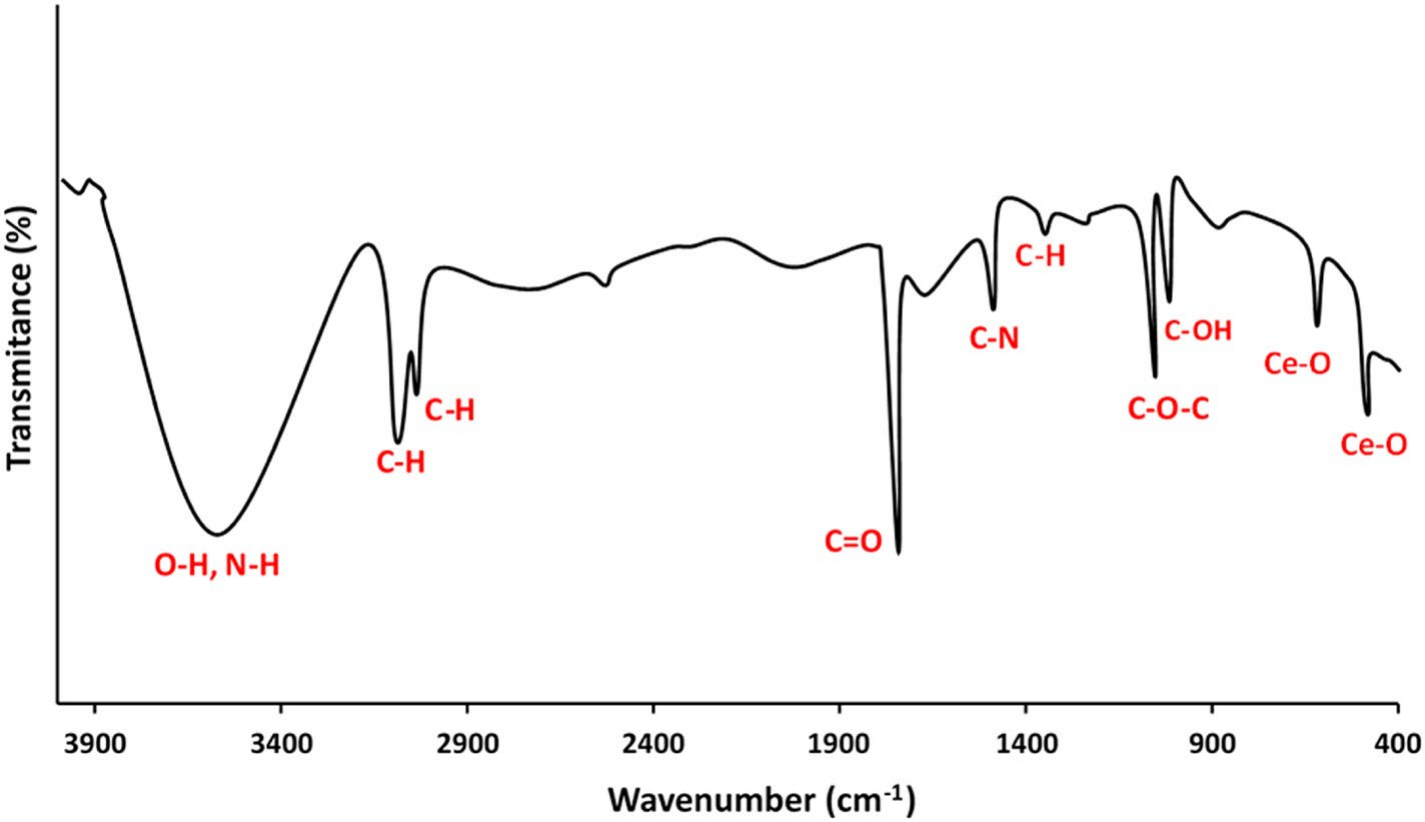
FTIR spectrum of the marine oyster assisted biosynthesized CeO_2_ NPs.

The surface chemistry of the sample was scrutinized using the FTIR technique. The related spectrum of bioprepared CeO_2_ NPs was illustrated in Fig. 6. The presence of several bands in the spectrum shows a wide range of marine oyster-derived biomolecules on the surface of nanoparticles. The strong and broad absorption peak at 3450 cm^−1^ is assigned to the plethora of hydroxyl groups, O‒H, and N‒H stretching due to water, carbohydrates, polyphenols, and probably proteins. The band at 2915 cm^−1^ is associated with aliphatic symmetric C‒H group stretching of carbohydrates. The characteristic vibration band of the carbonyl group (C=O) was observed at 1755 cm^−1^. The C–N stretch appears in the region 1400 cm^−1^. The weak C–H bending band of alkane compounds was appeared at 1365 cm^−1^. The vibration bands of ether functional group (C‒O‒C) linkage related to polysaccharides is centered at 1058 cm^−1^. The a quite strong stretching peak around 1000 cm^−1^ is related to C‒OH of primary and secondary alcoho^25^. The advent frequency bands at 745 cm^−1^ and 540 cm^−1^ are attributed to Ce‒O bonds stretching of bioproduced CeO_2_ NPs. The presence of copious phytochemicals in green reaction medium act a dual role as reducing and stabilizer agents of respective nanoceria^23^. As to the reduction process, this occurred through an electron transfer via redox reaction between electron-rich biomolecules (the reducing agent) as an electron donor and cerium cations as an electron acceptor (the oxidizing agent). Finally, the produced metallic cerium atoms upon exposure to the air oxygen would facilely produce CeO_2_ nanoparticles as reported in similar researches indicating biofabrication of nanoparticles via various biological derivatives^18,26^.

### Thermal gravimetric analysis (TGA) investigation

**Figure 7:**
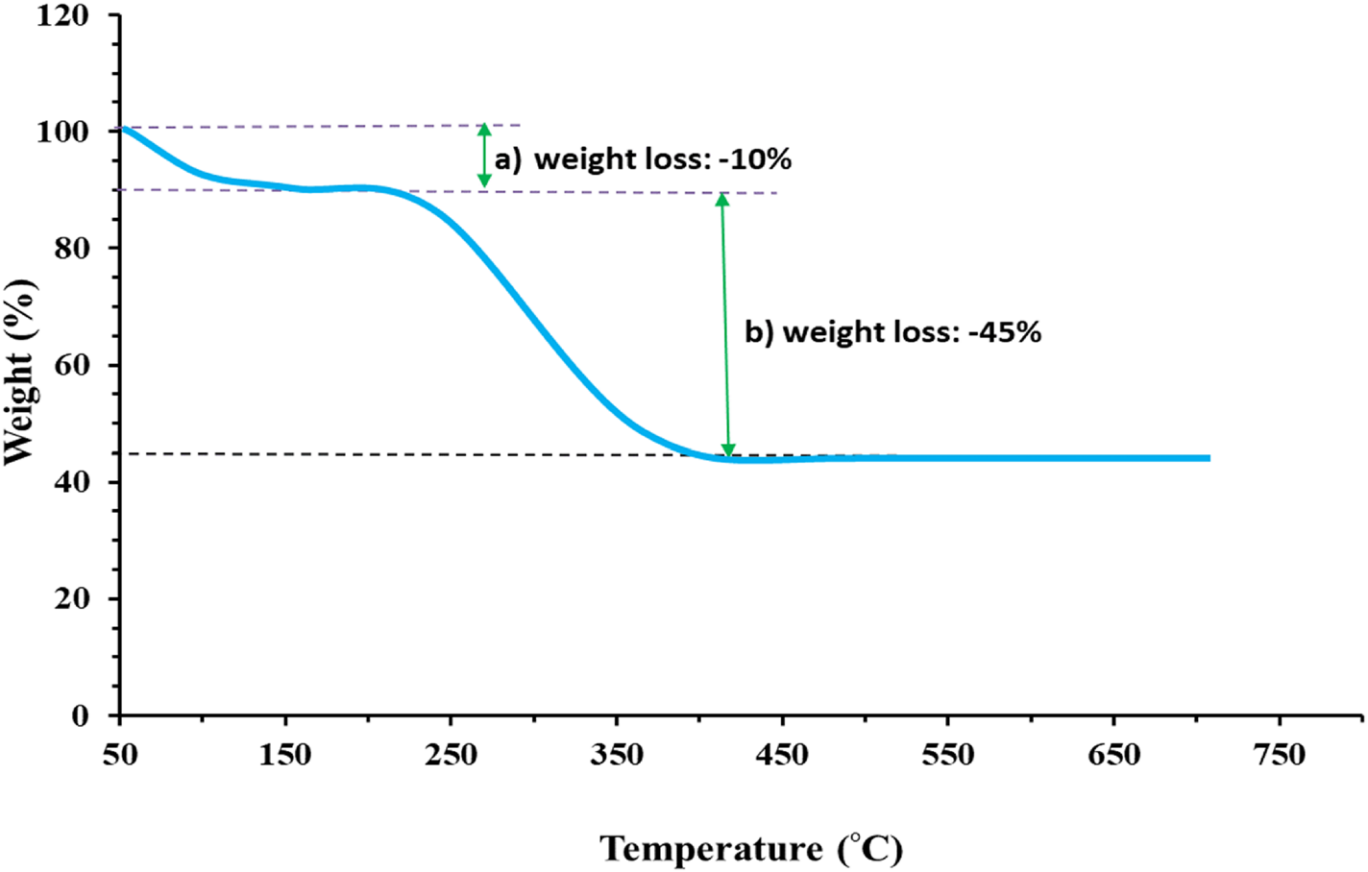
TGA plot of biofunctionalized CeO_2_ NPs using marine oyster extract.

The TGA curve of the biomolecules-capped nanoceria biofabricated using marine oyster extract in the temperature range of 100–700 °C is depicted in Fig. 7. TGA reveals two different weight loss percentages of the nanoceria specimen. The first degradation with 10% weight loss was detected in the temperature range of 50–200 °C likely owing to the evaporation of physically-adsorbed water molecules on the surface of CeO_2_ NPs. The second major mass change was appeared at temperature variation between 225 and 400 °C with 45% of the overall weight probably on account of desorption of marine-derived organic compounds in the nanoceria. Eventually, no reduction in the TGA plot was remarked, upon the further rise in the temperature indicating constant size maintenance of the sample. These results reveal the presence of marine-derived organic moieties on the surface of CeO_2_ NPs and hence confirm FTIR spectra findings.

### TEM and SEM characterization

**Figure 8:**
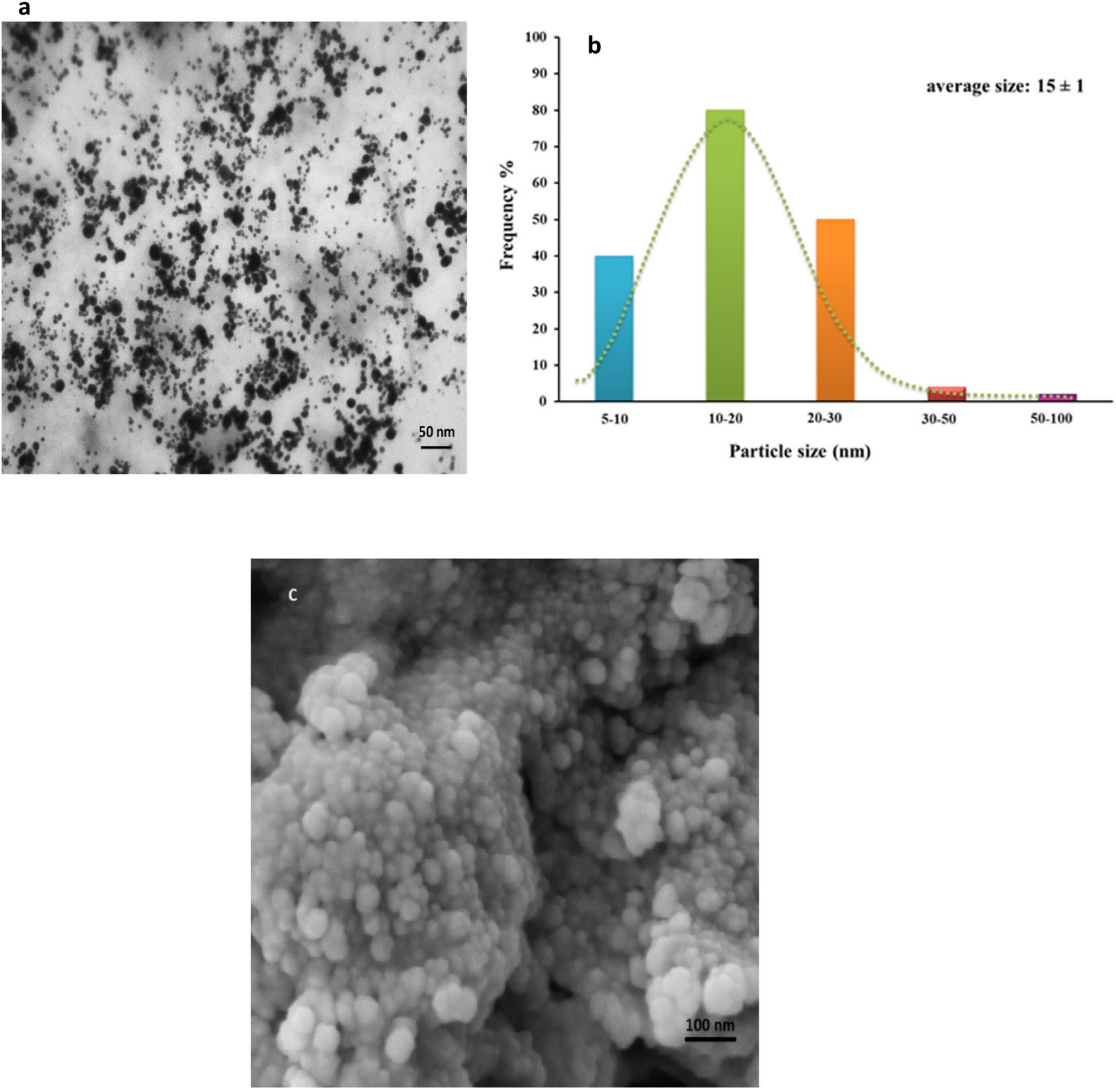
(**a**) TEM image, (**b**) particle size distribution histogram plot, and (**c**) SEM image of the marine oyster assisted biosynthesized CeO_2_ NPs.

TEM technique is powerful tool to determine the size and shape of designed nanoparticles. The TEM image of biological nanoceria using marine oyster extract is depicted in Fig. 8. It is found that biosynthesized CeO_2_ NPs are well dispersed and contained particles relatively spherical in shape. SEM characterization indicated that obtained product approximately possess dense nano-scale size particles with diameter range of 10–40 nm, fairly distributed in monodisperse fashion (Fig. 8c). The distribution particle size histogram obtained from the TEM micrograph analysis via ImageJ software exhibits an insignificant variation in particle size with a median diameter of 15 ± 1 nm. Likewise, plant extract-mediated biosynthesized nanoceria indicated spherically shaped geometry with a size of 10–70 nm mainly resulted from functional phenolic and flavonoids derived from *O. majorana L.* leaf extract^26^.

### Cytotoxic assay of green CeO_2_ NPs

**Figure 9:**
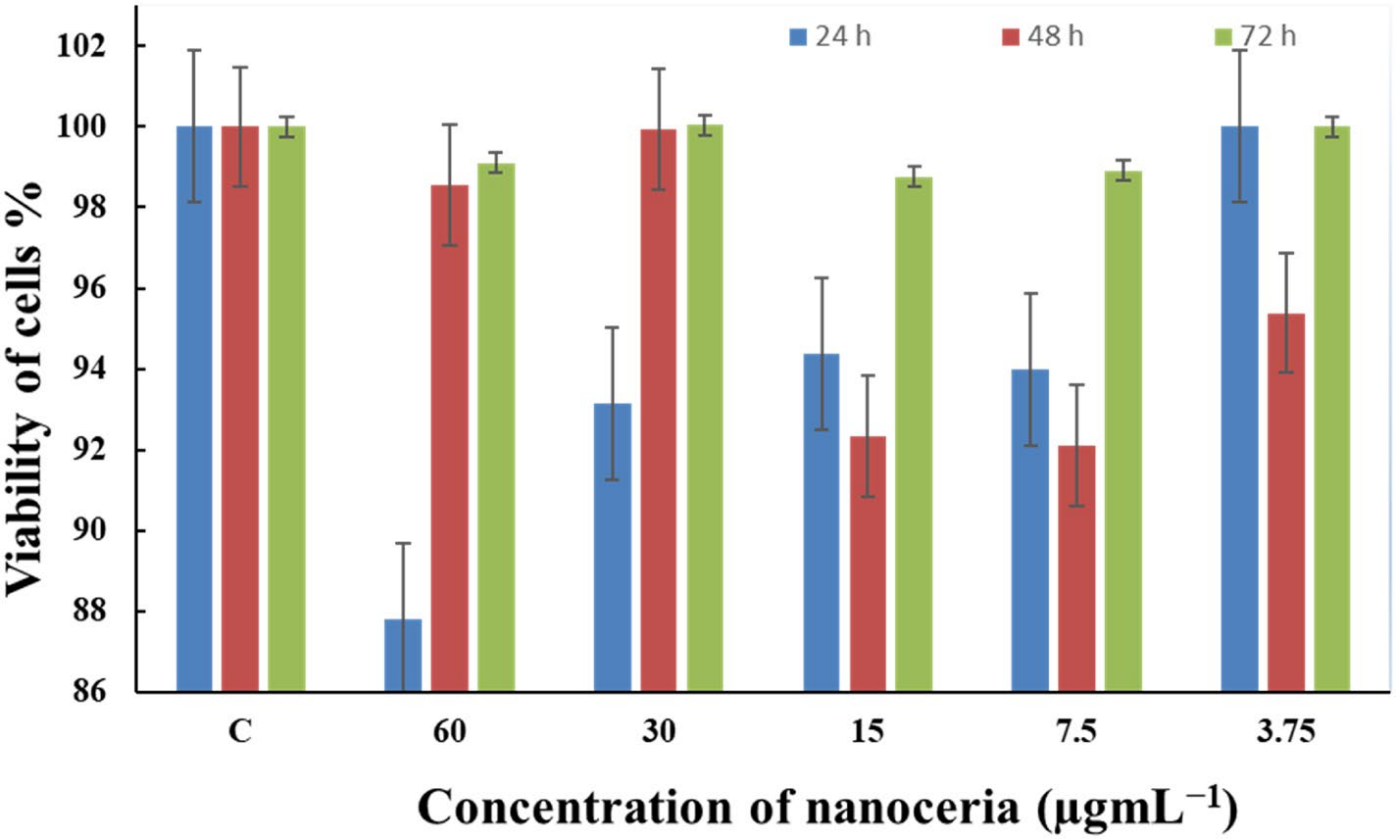
Cytotoxicity and biosafety of marine-mediated nanoceria (CeO_2_ NPs) against healthy L929 fibroblast cells.

To assess the potential cytotoxicity, we explored MTT assays to test the viability of L929 fibroblast cells subjected to concentrations of control (nanoceria-free) and CeO_2_ NPs at 3.75, 7.5, 15, 30, and 60 µg/mL and varying exposure time (24, 48, 72 h). As can be clearly seen in Fig. 9, the marine-mediated nanoceria are approximately nontoxic toward normal cell line indicating their biocompatibility and biosafety. Regardless of the nanoceria concentrations, the best result was observed at 72 h. In regard to exposure time, while the lowest percentage of cell viability was noticed at 48 h for 7.5 (92.09%) and 15 µg/mL (92.32%), respectively. However, the efficiency of marine-assisted CeO_2_ NPs are mainly concentration and time dependent and one is not able to address the constant trend signifying cytotoxicity manner of nanoparticles. The akin phenomena has been reported for green silver nanoparticles toward L929 cell line confirming our findings^27^. In a similar study also when rat cardiomyocytes (H9C2) and human brain fibroblast cells (T98G) were exposed to a 5 mg/mL dose of 30 nm CeO_2_, no major cytotoxicity was observed^28^. Being nontoxic nature of bio-prepared nanoceria could be highly likely attributed to the presence of substantial marine-derive biomolecules covered the surface of nanoparticle boosting their bioactivity and safety toward healthy cells as conformed with several literature reports^19,29^.

### Photocatalytic activity of CeO_2_ NPs

**Figure 10:**
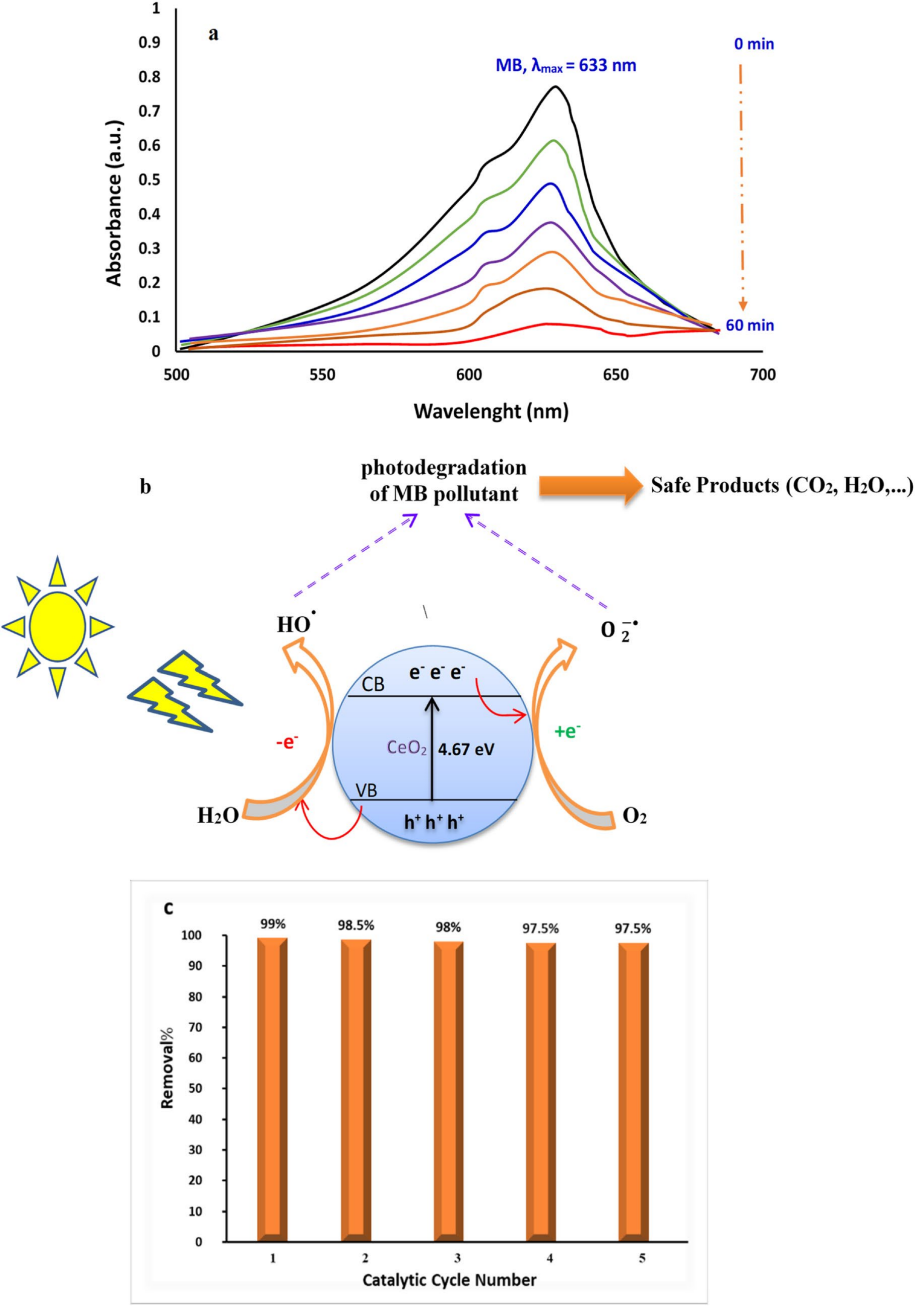
(**a**) Photocatalytic activity, (**b**) mechanism, and (**c**) recyclability of marine-mediated nanoceria against toward MB contaminant in aqueous solution.

**Figure 11:**
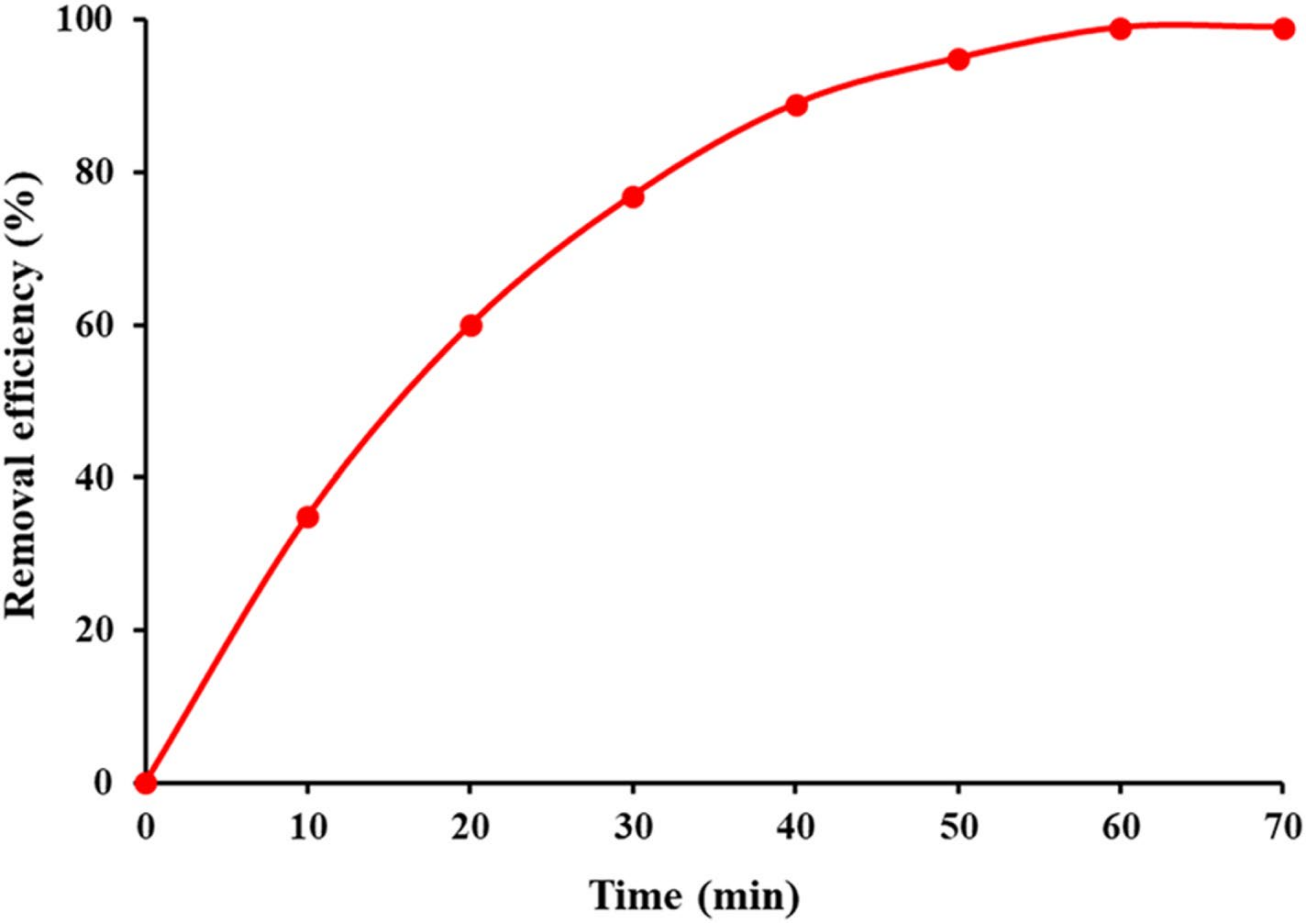
Mineralization and removal efficiency of MB dye as a function of time in the presence of CeO_2_ nanophotocatalyst with sunlight irradiation.

The catalytic efficiency of the biogenic CeO_2_ NPs was studied toward degradation of the organic methylthioninium chloride which is also known as Methylene Blue (MB). This heterocyclic aromatic compound is commonly utilized as day indicator in biochemistry and due to its redox properties has several medical applications including the treatment of methemoglobinemia, smooth muscle relaxation, near infrared fluorescence (NIR) fluorescent dye, and surgical procedure. Despite widely using MB as human therapeutic agent, its detrimental environmental impact has raised serious concerns^30^. The photodegradation was carried out in daylight using sunlight as a natural activator. It is found that by loading an appropriate dose (150 mg/L) of biological CeO_2_ NPs, the intensity of UV–Vis absorption peak of MB solution (150 ppm) at 633 nm was steadily disappeared within 60 min, thus demonstrating the rapid photocatalytic degradation of methylthioninium chloride contaminant from solution (Fig. 10a). It can be noted that optical band gap of energy acquired for green nanoceria is triggered by visible sunlight, and hence, production copious superoxide radicals during the action has piercing effect on accelerated deterioration of MB^31^ (Fig. 10b). The removal efficiency rate of MB dye was further monitored as function of time. It is found that 60 min is the time of choice for mineralization of MB organic pollutant as it can be seen in Fig. 11. Meanwhile, the reusability of CeO_2_ NPs photocatalyst toward MB degradation was also explored. After 5 consecutive cycles, no significant change was observed in the catalytic activity of nanoceria in removal rate of MB dye, indicating fairly high recyclability of biogenic CeO_2_ NPs (Fig. 10c). Further, The TEM investigation of lastly recycled CeO_2_ NPs was revealed no significant changes in morphology and particle size, exhibiting stability and reliability of biogenic ceria nanocatalyst (Fig. S1, see supplementary). Owing to outstanding photocatalytic properties, semiconductor photocatalysts are highly likely considered as competitive candidate on degradation organic contaminates in water and wastewater purification processes^32–37^. Moreover, several studies have been reported photodegradation of azo days using various nanomaterials such as CeO_2_ nanostructures^38^, CeO_2_/CuO nanocomposites^39^, Fe–doped CeO_2_ films^40^, CeO_2_/Fe_2_O_3_ composite^41^, phthalocyanine-TiO_2_ nanocomposite^42^, In/ZnO nanocomposite^43^ and V_2_O_5_-CeO_2_ nanocatalyst^44^. As per economic and environmental prospective, our findings show greater efficiency rather than chemically-produced CeO_2_ NPs toward same pollutant under UV irradiation^45^.

## Conclusion

In summary, we have developed a novel synthetic procedure for preparation of CeO_2_ NPs using marine oyster extract. Based on FTIR and TGA analyses the oyster extracted biomolecules function as bioreductant and stabilizing agent in green synthesis process of nanoceria. In the presence of electron-donor marine-derived biomolecules the metallic cations in aqueous salt solution were reduced into corresponding nanoceria in a benign and nontoxic reaction condition. XRD, TEM, and SEM results confirmed the cubic fluorite structure of biogenic CeO_2_ NPs without any impurities with a mean particle size of 15 ± 1 nm distributed in homogenous fashion. During the photocatalysis process, the superoxide radicals generated by nanoceria are the dominant oxidants in MB elimination process. The results show that biogenic CeO_2_ NPs are highly recyclable semiconductor catalyst toward degradation of MB pollutant in aqueous solution. Further, the marine-mediated nanoceria show higher level of biological safety as no major cytotoxic effect has been noticed against cell line using MTT method. The designed synthetic route presents great advantages including simplicity, biocompatibility, and high potency of large-scale production of nanomaterials.

## Methods

All chemical reagents were obtained from Sigma-Aldrich and used without further purifications.

### Oyster extract preparation

The native raw Oysters (*Ostreidae*) species were collected from the rocky shores of Chabahar, Iran. Freshly shucked oyster muscles were completely washed using the tap and distilled water to remove any sediments and other foreign substances. the fleshes then were chopped into small bits, packed in aluminum foils, and freeze dried for 72 h at − 80 °C. The dried specimens were then powdered by means of a mechanical mixer for 5 min and properly sieved to a desirable particle size through a stainless-steel sieve of the 9-mesh screen. Subsequently, ground sample material of 5 g was heated into a backer containing 200 mL of methanol at a temperature of 80 °C for 120 min. Finally, rotary evaporator extraction was performed at 40 °C to concentrate extract and evaporate the extra solvent.

### Biosynthesis of CeO_2_ NPs

An aliquot of 0.5 g of Cerium(III) nitrate hexahydrate [Ce (NO_3_)_3_·6H_2_O] was weighed and added to 100 mL of methanolic oyster extract solution. Then the solution was mixed and heated in a water bath for 2 h under constant stirring at 150 rpm. In order to obtain homogeneous dispersion, the mixture further was subject to ultrasonic treatment performed at 150 W power and 35 kHz frequency for 10 min. Once the resultant solution cooled down to room temperature a pale-yellow solid precipitate was attained. The ensuing solid was separated from the supernatant using constant centrifugation at 15,000 rpm for 5 min. The precipitate was then dried at 95 °C overnight in an oven to remove additional impurities.

### Cytotoxicity experiment of biological nanoceria

For the evaluation of the cytotoxic effect and biocompatibility of bioprepared CeO_2_ NPs, MTT (3-Dimethylthiazo-2,5-diphynyltetrazolium Bromide) assay was used on L929 cell line following instruction from the manufacturer and literature^46^. L929 is a normal fibroblast cell line from subcutaneous connective tissue of mouse of which their cultures were incubated at a density of 1 × 105 cells per well at 37 °C in 5% CO_2_. CeO_2_ NPs were added in different concentrations, and after 24 h, the cells were washed twice with phosphate buffer saline (PBS) before adding fresh 100 µL of culture medium and 0.5 mg/mL of MTT reagent to each well. The control group served as the non-labeled cells. Subsequently, the labeled cells were incubated at 37 °C in 5% CO_2_ for 4 h, the medium was kindly aspired and replaced by 100 μg/mL of fresh DMSO. Dissolved formazan product absorbance was measured at a wavelength of 570 nm. The mean optical density (OD, absorbance) of four wells in the indicated groups was employed to measure the percentage of cell viability as below:

$$ Percentage  \, of \,  cell \,  viability \, = \, \left( {A_{Sample} /A_{Control} } \right) \, \times 100 $$

where *A*_Sample_ was average OD reading of different incubated treated cells of both cell lines and *A*_Control_ was average OD reading of the different incubated cells in complete culture media only. The cytotoxicity of the cells was then assessed from the average triplicate values and exhibited as mean ± standard deviation (SD).

### Catalytic properties of biogenic nanoceria

The photocatalytic activity of CeO_2_ NPs for degradation of organic methylene blue (MB) as biomedical day agent was explored in visible region under sunlight irradiation based on literature report^14^. In so doing, in a transparent approximately flat beaker, a 150 mg of nanoceria is well dispersed in 150 ppm of respective MB under optimized conditions. All the experiments of samples were inspected for a period of 60 min. A 10 mL sample was taken each 10 min interval and the photodegradation process was monitored using UV–Vis spectrophotometer at the maximum absorption wavelength of MB (633 nm).

### Characterization of biomediated CeO_2_ NPs

The biosynthesized CeO_2_ NPs were systematically studied using a litany of characterization techniques using UV/vis spectrophotometer (Shimadzu UV-2550, Japan), FTIR (Shimadzu 8400S, Japan), XRD (PANalytic X'Pert MPD, PANalytical, Almelo, Netherlands), FESEM (Zeiss Sigma 500 VP, Germany), and TEM (Zeiss-EM10C-100 kV, Germany). Raman (Takram P50C0R10, laser wavelength = 532 nm), and thermogravimetric analysis (TGA, PerkinElmer, Pyris 1, USA).

## Supplementary Information

Supplementary Information.
