# Supplementary material for: Enhanced sunlight photocatalytic activity and biosafety of marine-driven synthesized cerium oxide nanoparticles
Source: Sci Rep. 2021 Jul 19;11:14734. doi: 10.1038/s41598-021-94327-w (PMC8289931; doi:10.1038/s41598-021-94327-w)
Supplement: Supplementary file 1 — Supplementary Information. [file 41598_2021_94327_MOESM1_ESM.pdf]

Enhanced Sunlight Photocatalytic Activity and Biosafety of Marine-Driven Synthesized Cerium Oxide Nanoparticles

Somayeh Safat[a], Foad Buazar[a*], Salim Albukhaty[b], Soheila Matroodi[c]

[a]*Department of Marine Chemistry, Khorramshahr University of Marine Science and Technology, P.O. Box 669, Khorramshahr, Iran*
[b]*Department of Chemistry, University of Misan, P.O. Box 62001, Maysan, Iraq*
[c]*Department of Marine Biology, Khorramshahr University of Marine Science and Technology, P.O. Box 669, Khorramshahr, Iran*

[*] *Corresponding author, Tel: +98-916115084, Email: fb@kmsu.ac.ir*

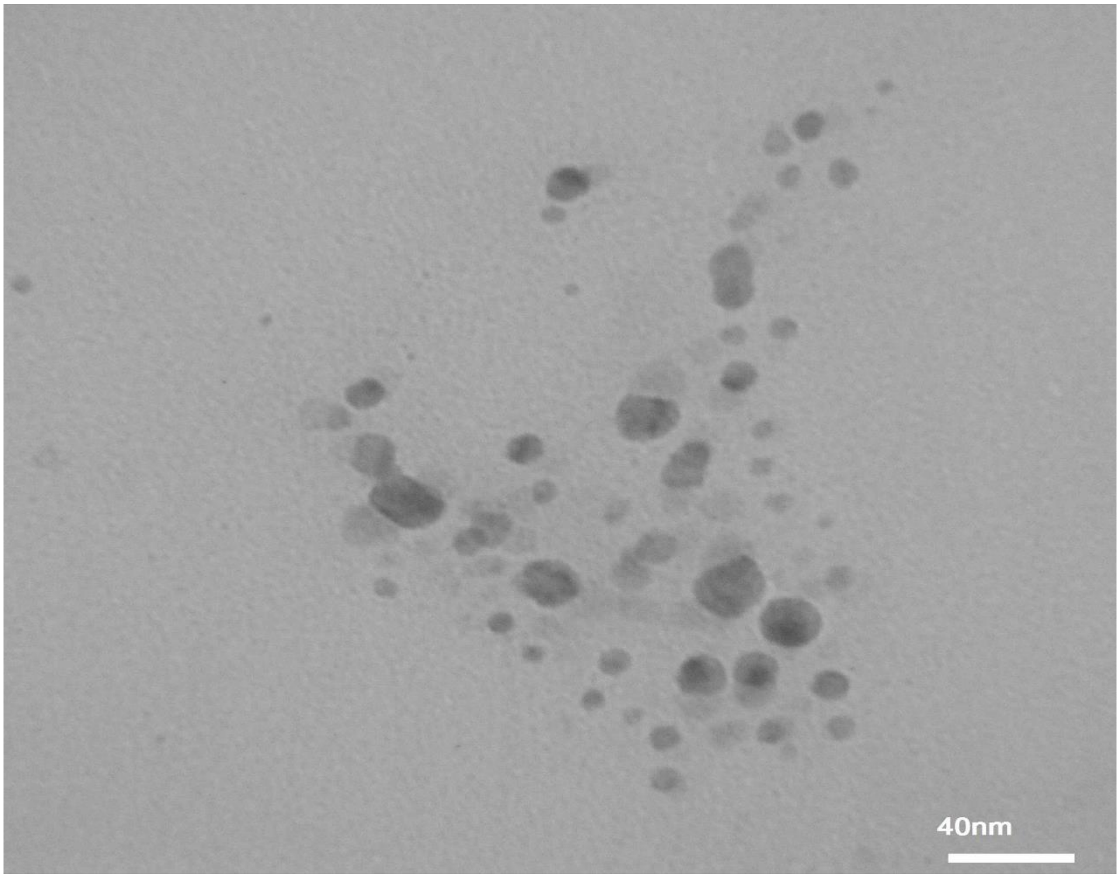

Figure S1. TEM image of recycled biogenic CeO_2 NPs
